# Supplementary material for: Changes in physical activity levels, eating habits and psychological well-being during the Italian COVID-19 pandemic lockdown: Impact of socio-demographic factors on the Florentine academic population
Source: PLoS One. 2021 May 27;16(5):e0252395. doi: 10.1371/journal.pone.0252395 (PMC8159001; doi:10.1371/journal.pone.0252395)
Supplement: S2 File — (DOCX) [file pone.0252395.s002.docx]

**S2 File. ABITUD-19 Questionnaire**

Questionnaire related to changes in habits induced by the COVID-19 quarantine.

This study investigates the impact of quarantine on daily habits. Each participant will have to answer questions regarding physical activity, nutrition and emotions related to periods both before and during the quarantine imposed by the COVID-19 pandemic.

**General Information:**

- Age:
- Gender:

Male or Female

- Marital status:

Unmarried/maiden, Married / cohabiting, Widower, Separated or divorced

- Education:

Elementary School, Middle School, High School, Degree, PhD

- Work before quarantine:

Permanent employee, Fixed-term employee, Self-employed, On-call contract, Training / internship, Student, unemployed, Retired, Other

- Work during quarantine:

Remote work, Health worker, Essential service worker, Self-employed without activity, Redundancy fund, Licensed, Volunteer, Retired, Other

- House size:

<50 square meters, 50-80 square meters, 80-100 square meters,> 100 square meters

- Number of inhabitants during the quarantine:

Alone, 2, 3, 4, >4

- Presence of open spaces at home:

Habitable terrace, Garden, No

- Have you had the loss of a loved one due to COVID-19?

YES, NO

**Eating habits BEFORE quarantine**

1. Weight in kg (before quarantine)
2. Height in cm
3. Where did you have breakfast?

Home, Canteen / Office, Bar / Fast Food / Diner, Pizzeria / Restaurant, I don't do it, other

1. Where did you have mid-morning snack?

Home, Canteen / Office, Bar / Fast Food / Diner, Pizzeria / Restaurant, I don't do it, other

1. Where did you have lunch?

Home, Canteen / Office, Bar / Fast Food / Diner, Pizzeria / Restaurant, I don't do it, other

1. Where did you have afternoon snack?

Home, Canteen / Office, Bar / Fast Food / Diner, Pizzeria / Restaurant, I don't do it, other

1. Where did have dinner?

Home, Canteen / Office, Bar / Fast Food / Diner, Pizzeria / Restaurant, I don't do it, other

1. How often did you consume unrefined grains (whole meal bread, pasta, rice ...) in a week?

Never, 1-6, 7-12, 13-18, 19-31, > 32

1. How often did you eat potatoes in a week?

Never, 1-4, 5-8, 9-12, 13-18,> 18

1. How often did you consume fruit in a week?

Never, 1-4, 5-8, 9-15, 16-21,> 22

1. How often did you eat the vegetables in a week?

Never, 1-6, 7-12, 13-20, 21-32,> 33

1. How often did you consume legumes in a week?

Never, 1, 1-2, 3-4, 5-6,> 6

1. How often did you consume the fish in a week?

Never, 1, 1-2, 3-4, 5-6,> 6

1. How often did you eat red meat in a week?

Never, 1, 2-3, 4-5, 6-7, <8

1. How often did you eat white meat in a week?

Never, 2-3, 4-5, 6-7, 8-10,> 10

1. How often did you consume dairy products (cheese, milk, and yogurt) in a week?

<10, 11-15, 16-20, 21-28, 29-30,> 30

1. How many times a week did you use cooking olive oil in a week?

Never, Rarely, <1, 1-3, 3-5, Daily

1. How many ml of alcoholic beverages did you consume per day?

<300, 300, 400, 500, 600,> 700

**Eating habits DURING quarantine**

1. Weight in kg (before quarantine)
2. Where do you have breakfast?

Home, Canteen / Office, Bar / Fast Food / Diner, Pizzeria / Restaurant, I don't do it, other

1. Where do you have mid-morning snack?

Home, Canteen / Office, Bar / Fast Food / Diner, Pizzeria / Restaurant, I don't do it, other

1. Where do you have lunch?

Home, Canteen / Office, Bar / Fast Food / Diner, Pizzeria / Restaurant, I don't do it, other

1. Where do you have afternoon snack?

Home, Canteen / Office, Bar / Fast Food / Diner, Pizzeria / Restaurant, I don't do it, other

1. Where do you have dinner?

Home, Canteen / Office, Bar / Fast Food / Diner, Pizzeria / Restaurant, I don't do it, other

1. How often do you consume unrefined grains (whole meal bread, pasta, rice ...) in a week?

Never, 1-6, 7-12, 13-18, 19-31, > 32

1. How often do you eat potatoes in a week?

Never, 1-4, 5-8, 9-12, 13-18,> 18

1. How often do you consume fruit in a week?

Never, 1-4, 5-8, 9-15, 16-21,> 22

1. How often do you eat the vegetables in a week?

Never, 1-6, 7-12, 13-20, 21-32,> 33

1. How often do you consume legumes in a week?

Never, 1, 1-2, 3-4, 5-6,> 6

1. How often do you consume the fish in a week?

Never, 1, 1-2, 3-4, 5-6,> 6

1. How often do you eat red meat in a week?

Never, 1, 2-3, 4-5, 6-7, <8

1. How often do you eat white meat in a week?

Never, 2-3, 4-5, 6-7, 8-10,> 10

1. How often do you consume dairy products (cheese, milk and yogurt) in a week?

<10, 11-15, 16-20, 21-28, 29-30,> 30

1. How many times a week do you use cooking olive oil in a week?

Never, Rarely, <1, 1-3, 3-5, Daily

1. How many ml of alcoholic beverages do you consume per day?

<300, 300, 400, 500, 600,> 700

**Physical activity BEFORE quarantine**

1. Did your work involve vigorous-intensity activity that causes large increases in breathing or heart rate like [carrying or lifting heavy loads, digging or construction work] for at least 10 minutes continuously?

YES, NO If No, go to P 4

1. In a typical week, on how many days did you do vigorous intensity activities as part of your work?

0, 1, 2, 3, 4, 5, 6, 7

1. How much time did you spend doing vigorous-intensity activities at work on a typical day?

Hours: minutes

1. Did your work involve moderate-intensity activity, that causes small increases in breathing or heart rate such as brisk walking (or carrying light loads) for at least 10 minutes continuously?

YES, NO If No, go to P 7

1. In a typical week, on how many days did you do moderate intensity activities as part of your work?

0, 1, 2, 3, 4, 5, 6, 7

1. How much time did you spend doing moderate-intensity activities at work on a typical day?

Hours: minutes

1. The next questions exclude the physical activities at work that you have already mentioned. Now I would like to ask you about the usual way you travel to and from places. For example to work, for shopping, to market, to place of worship. Did you walk or use a bicycle (pedal cycle) for at least 10 minutes continuously to get to and from places?

YES, NO If No, go to P 10

1. In a typical week, on how many days did you walk or bicycle for at least 10 minutes continuously to get to and from places?

0, 1, 2, 3, 4, 5, 6, 7

1. How much time did you spend walking or bicycling for travel on a typical day?

Hours : minutes

1. The next questions exclude the work and transport activities that you have already mentioned. Now I would like to ask you about sports, fitness and recreational activities (leisure). Did you do any vigorous-intensity sports, fitness or recreational (leisure) activities that cause large increases in breathing or heart rate like [running or football] for at least 10 minutes continuously?

YES, NO If No, go to P 13

1. In a typical week, on how many days did you do vigorous intensity sports, fitness or recreational (leisure) activities?

0, 1, 2, 3, 4, 5, 6, 7

1. How much time did you spend doing vigorous-intensity sports, fitness or recreational activities on a typical day?

Hours: minutes

1. Did you do any moderate-intensity sports, fitness or recreational (leisure) activities that cause a small increase in breathing or heart rate such as brisk walking, (cycling, swimming, and volleyball) for at least 10 minutes continuously?

YES, NO If No, go to P 16

1. In a typical week, on how many days did you do moderate intensity sports, fitness or recreational (leisure) activities?

0, 1, 2, 3, 4, 5, 6, 7

1. How much time did you spend doing moderate-intensity sports, fitness or recreational (leisure) activities on a typical day?

Hours: minutes

1. Sedentary behaviors. The following question is about sitting or reclining at work, at home, getting to and from places, or with friends including time spent sitting at a desk, sitting with friends, traveling in car, bus, train, reading, playing cards or watching television, but do not include time spent sleeping. How much time did you usually spend sitting or reclining on a typical day?

Hours: minutes

**Physical activity DURING quarantine**

1. Does your work involve vigorous-intensity activity that causes large increases in breathing or heart rate like [carrying or lifting heavy loads, digging or construction work] for at least 10 minutes continuously?

YES, NO If No, go to P 4

1. In a typical week, on how many days do you do vigorous intensity activities as part of your work?

0, 1, 2, 3, 4, 5, 6, 7

1. How much time do you spend doing vigorous-intensity activities at work on a typical day?

Hours: minutes

1. Does your work involve moderate-intensity activity, that causes small increases in breathing or heart rate such as brisk walking (or carrying light loads) for at least 10 minutes continuously?

YES, NO If No, go to P 7

1. In a typical week, on how many days do you do moderate intensity activities as part of your work?

0, 1, 2, 3, 4, 5, 6, 7

1. How much time do you spend doing moderate-intensity activities at work on a typical day?

Hours: minutes

1. The next questions exclude the physical activities at work that you have already mentioned. Now I would like to ask you about the usual way you travel to and from places. For example to work, for shopping, to market, to place of worship. Do you walk or use a bicycle (pedal cycle) for at least 10 minutes continuously to get to and from places?

YES, NO If No, go to P 10

1. In a typical week, on how many days do you walk or bicycle for at least 10 minutes continuously to get to and from places?

0, 1, 2, 3, 4, 5, 6, 7

1. How much time do you spend walking or bicycling for travel on a typical day?

Hours: minutes

1. The next questions exclude the work and transport activities that you have already mentioned. Now I would like to ask you about sports, fitness and recreational activities (leisure). Do you do any vigorous-intensity sports, fitness or recreational (leisure) activities that cause large increases in breathing or heart rate like [running or football] for at least 10 minutes continuously?

YES, NO If No, go to P 13

1. In a typical week, on how many days do you do vigorous intensity sports, fitness or recreational (leisure) activities?

0, 1, 2, 3, 4, 5, 6, 7

1. How much time do you spend doing vigorous-intensity sports, fitness or recreational activities on a typical day?

Hours: minutes

1. Do you do any moderate-intensity sports, fitness or recreational (leisure) activities that cause a small increase in breathing or heart rate such as brisk walking, (cycling, swimming, and volleyball) for at least 10 minutes continuously?

YES, NO If No, go to P 16

1. In a typical week, on how many days do you do moderate intensity sports, fitness or recreational (leisure) activities?

0, 1, 2, 3, 4, 5, 6, 7

1. How much time do you spend doing moderate-intensity sports, fitness or recreational (leisure) activities on a typical day?

Hours: minutes

1. Sedentary behaviors. The following question is about sitting or reclining at work, at home, getting to and from places, or with friends including time spent sitting at a desk, sitting with friends, traveling in car, bus, train, reading, playing cards or watching television, but do not include time spent sleeping. How much time do you usually spend sitting or reclining on a typical day?

Hours: minutes

**Psychological well-being BEFORE quarantine**

- Were you generally tense or did you feel any tension before quarantine?

Yes, extremely tense, most or all of the time

Yes, very tense most of the time

Not generally tense, but did feel fairly tense several times

I felt a little tense a few times

My general tension level was quite low

I never felt tense or any tension at all

- Did you feel depressed before quarantine?

Yes, to the point that I felt like taking my life

Yes, to the point that I did not care about anything

Yes, very depressed almost every day

Yes, quite depressed several times

Yes, a little depressed now and then

No, never felt depressed at all

- I felt cheerful, lighthearted before quarantine.

None of the time

A little of the time

Some of the time

A good bit of the time

Most of the time

All of the time

- Have you been in firm control of your behavior, thoughts, emotions or feelings before quarantine?

Yes, definitely so

Yes, for the most part

Generally so

Not too well

No, and I am somewhat disturbed

No, and I am very disturbed

- Did you feel healthy enough to carry out the things you like to do or had to do before quarantine?

Yes, definitely so

For the most part

Health problems limited me in some important ways

I was only healthy enough to take care of myself

I needed some help in taking care of myself

I needed someone to help me with most or all of the things I had to do

- How much energy, pep, or vitality did you have or feel before quarantine?

Very full of energy, lots of pep

Fairly energetic most of the time

My energy level varied quite a bit

Generally low in energy or pep

Very low in energy or pep most of the time

No energy or pep at all, I fell drained, sapped

**Psychological well-being DURING quarantine**

- Are you generally tense or do you feel any tension during quarantine?

Yes, extremely tense, most or all of the time

Yes, very tense most of the time

Not generally tense, but did feel fairly tense several times

I felt a little tense a few times

My general tension level was quite low

I never felt tense or any tension at all

- Do you feel depressed during quarantine?

Yes, to the point that I felt like taking my life

Yes, to the point that I did not care about anything

Yes, very depressed almost every day

Yes, quite depressed several times

Yes, a little depressed now and then

No, never felt depressed at all

- I fell cheerful, lighthearted during quarantine.

None of the time

A little of the time

Some of the time

A good bit of the time

Most of the time

All of the time

- Are you in firm control of your behavior, thoughts, emotions or feelings during quarantine?

Yes, definitely so

Yes, for the most part

Generally so

Not too well

No, and I am somewhat disturbed

No, and I am very disturbed

- Do you feel healthy enough to carry out the things you like to do or have to do during quarantine?

Yes, definitely so

For the most part

Health problems limited me in some important ways

I was only healthy enough to take care of myself

I needed some help in taking care of myself

I needed someone to help me with most or all of the things I had to do

- How much energy, pep, or vitality do you have or feel during quarantine?

Very full of energy, lots of pep

Fairly energetic most of the time

My energy level varied quite a bit

Generally low in energy or pep

Very low in energy or pep most of the time

No energy or pep at all, I fell drained, sapped
