# Supplementary material for: Changes in physical activity levels, eating habits and psychological well-being during the Italian COVID-19 pandemic lockdown: Impact of socio-demographic factors on the Florentine academic population
Source: PLoS One. 2021 May 27;16(5):e0252395. doi: 10.1371/journal.pone.0252395 (PMC8159001; doi:10.1371/journal.pone.0252395)
Supplement: Attachment — Submitted filename: Revised Manuscript Review.docx [file pone.0252395.s004.docx]

Revised Manuscript Review

Manuscript Number: PONE-D-21-10183R1

Line 29: the aim of the study was to identify changes in psychological well being and relationship with socio-demographic parameters.

Line 36: change were to was

Line 44-45: can be considered factors for the changes……(delete the word risk)

Line 148-149 (not sure if the words Metabolic Equivalents need to be capitalized)

Line 225: Figure 2 shows the place where meals were consumed.

Line 226: The comparison between pre and post-quarantine shows………..

**Discussion Section:**

This needs to be re-written for clarity.

*Because an increase in obesity has been was hypothesized following the pandemic, with a consequent increase in cardiovascular risk, the scientific For preventive purposes, the literature was had therefore geared towards promoted preventive measures such as healthy eating and physical activity at home during the quarantine period [15, 16].*

*However, psychological well-being appears to be impaired mainly in people of*

*affected by young age and when working conditions change.*

Consider changing to

*However, psychological well-being appears to be impaired mainly in young people of*

*when working conditions change.*
