# Supplementary material for: Changes in physical activity levels, eating habits and psychological well-being during the Italian COVID-19 pandemic lockdown: Impact of socio-demographic factors on the Florentine academic population
Source: PLoS One. 2021 May 27;16(5):e0252395. doi: 10.1371/journal.pone.0252395 (PMC8159001; doi:10.1371/journal.pone.0252395)
Supplement: Attachment — Submitted filename: Response to Reviewers.docx [file pone.0252395.s005.docx]

Review Comments to the Author

Dear Editor and Reviewers, thank you for the work done in order to improve our manuscript.

The response letter to the reviewers was made after each point-by-point observation. Responses are highlighted in red.

Reviewer #1: There are a few minor language issues but the revised manuscript is much stronger and ready for publication.

Line 29: the aim of the study was to identify changes in psychological well being and relationship with socio-demographic parameters.

Thank you for the proposal, corrections have been made accordingly.

Line 36: change were to was

Thank you for the proposal, corrections have been made accordingly.

Line 44-45: can be considered factors for the changes……(delete the word risk)

Thank you for the proposal, corrections have been made accordingly.

Line 148-149 (not sure if the words Metabolic Equivalents need to be capitalized)

In order to provide the reader with an overall definition of the abbreviation, the authors prefer to leave it in its entirety by including also the term TASK that completes the abbreviation MET.

Line 225: Figure 2 shows the place where meals were consumed.

Thank you for the proposal, corrections have been made accordingly.

Line 226: The comparison between pre and post-quarantine shows………..

Thank you for the proposal, corrections have been made accordingly.

**Discussion Section:**

This needs to be re-written for clarity.

*Because an increase in obesity has been was hypothesized following the pandemic, with a consequent increase in cardiovascular risk, the scientific For preventive purposes, the literature was had therefore geared towards promoted preventive measures such as healthy eating and physical activity at home during the quarantine period [15, 16].*

Thank you for the proposal, the actual sentence is: “An increase in obesity was hypothesized following the pandemic, with a consequent increase in cardiovascular risk. For preventive purposes, the literature had therefore promoted healthy eating and physical activity at home during the quarantine period.”

*However, psychological well-being appears to be impaired mainly in people of*

*affected by young age and when working conditions change.*

Consider changing to

*However, psychological well-being appears to be impaired mainly in young people of*

*when working conditions change.*

Thank you for the proposal, the actual sentence is: “However, psychological well-being appears to be impaired mainly in young people and when working conditions change.”

Reviewer #2: Dear Prof. Dr. Cristina Cortis,

thank you very much for the possibility to re-serve as a reviewer in a prestigious periodical like Plos One.

Also, I would like to commend the authors for their revision work. There is only one minor request, that I would like to reiterate. I think that adds value to the study.

The authors answered my question about response rate by emphasizing the use of three validated questionaries. This certainly shows the validity of the work, but I believe that what the authors reported in their reply to my comment should also be reported in the text at Line 176. I re-suggest to add a possible rationale about this data.

Thank you for the proposal. As suggest by reviewer, a sentence has been added at line 173, just before the version of the software used for statistical analysis:

“Inference based on selected samples – e.g. due to low response rate – is valid provided variables related to the selection mechanism are identified and included as confounders in the analysis [15]. The multiple regression analysis performed on the outcome variables adjusts for individual propensity, which is the most plausible determinants of the response rate.”
